# Multiplex Detection of Different Magnetic Beads Using Frequency Scanning in Magnetic Frequency Mixing Technique

**DOI:** 10.3390/s19112599

**Published:** 2019-06-07

**Authors:** Stefan Achtsnicht, Ali Mohammad Pourshahidi, Andreas Offenhäusser, Hans-Joachim Krause

**Affiliations:** 1 Institute of Complex Systems Bioelectronics (ICS-8), Forschungszentrum Jülich, 52425 Jülich, Germany; s.achtsnicht@fz-juelich.de (S.A.); a.pourshahidi@fz-juelich.de (A.M.P.); a.offenhaeusser@fz-juelich.de (A.O.); 2 RWTH Aachen University, 52062 Aachen, Germany

**Keywords:** frequency mixing magnetic detection, magnetic sandwich immunoassay, multiparametric immunoassays

## Abstract

In modern bioanalytical methods, it is often desired to detect several targets in one sample within one measurement. Immunological methods including those that use superparamagnetic beads are an important group of techniques for these applications. The goal of this work is to investigate the feasibility of simultaneously detecting different superparamagnetic beads acting as markers using the magnetic frequency mixing technique. The frequency of the magnetic excitation field is scanned while the lower driving frequency is kept constant. Due to the particles’ nonlinear magnetization, mixing frequencies are generated. To record their amplitude and phase information, a direct digitization of the pickup-coil’s signal with subsequent Fast Fourier Transformation is performed. By synchronizing both magnetic fields, a stable phase information is gained. In this research, it is shown that the amplitude of the dominant mixing component is proportional to the amount of superparamagnetic beads inside a sample. Additionally, it is shown that the phase does not show this behaviour. Excitation frequency scans of different bead types were performed, showing different phases, without correlation to their diverse amplitudes. Two commercially available beads were selected and a determination of their amount in a mixture is performed as a demonstration for multiplex measurements.

## 1. Introduction

Magnetic beads (MBs) are often applied in modern bioanalytical and biomedical applications where they are used as handles or carriers or both [1,2,3]. In their function as labels, they can, for example, be used in an immunological method to detect a target substance inside a sample. Such targets can be bacteria, cells, spores, viruses or toxins [4]. These methods have in common that they are usually relatively fast, cost-effective and robust, without the need for extensive sample preparation [5]. Usually, a specific capture part—which can be an antibody, a phage, a nucleic acid (Aptamer), a molecular imprinted polymer or similar [5]—is bound to the magnetic bead label. Different magnetic field sensors are available to detect and quantify the amount of MBs inside a sample, for example, Hall sensors [6], Faraday coils, giant magnetoresistance (GMR) [7] sensors and superconducting quantum interference devices (SQUIDS) [8]. Based on these different sensors, a broad variety of techniques have been established, for example, relaxometry [9], susceptometry [10] and nuclear magnetic resonance [11]. In this work, we used the magnetic frequency mixing technique [12,13] to detect the magnetic beads. This technique has the advantage of being very selective to superparamagnetic particles. It has been successfully applied for the detection of numerous different biological targets [14,15,16].

When using MBs as labels, it would often be desirable to detect multiple targets in one measurement run, for example, during the diagnosis of a certain disease [17,18]. Different approaches have been performed both experimentally and in simulations to realize so-called multiparametric immunoassays [19]. Recently, we have shown a method where this is done by using the same MBs but with spatial separation of different capture zones [20]. When different types of MBs are used to label different targets, their different magnetic properties can be used to distinguish them without the need of spatial separation. In Reference [21], a method has been suggested to distinguish MBs based on their behaviour in different magnetic bias fields. For this purpose, Monte Carlo simulations of free moving particles were used under conditions as they are common during magnetic particle imaging (MPI). Another research investigated the separation in magnetorelaxometry (MRX) techniques based on different particle types’ relaxation time behaviour [22]. In the simulation study by Wu et al. [23], using the magnetic frequency mixing technique, the distinction of the type of beads is based on the different magnetization behaviour of their magnetic core material. MBs have basically two relaxation mechanisms (Néel and Brown). Which is the dominant one depends amongst other on the (hydrodynamic) size of the MBs. In References [24,25], two different types of MBs were used, one with a hydrodynamic size of about 25 nm with dominant Brownian relaxation, while the other one, which was only 10 or 12 nm in diameter, was Néel dominated. They mention that for their type, the crossover between both mechanisms occurs at diameters around 20 nm. Their results show that it works well when both MBs’ relaxations are chosen such that they relax via different relaxation mechanisms.

In this research, we analysed whether different MB types can be distinguished by using the magnetic frequency mixing technique and by scanning the frequency of the magnetic probing field. If this is possible, we can later use this to develop an immunoassay where different targets can be detected and quantified in the same sample at the same time without the need of any mechanical movement of the sample holder, as it was done in Reference [20]. In contrast to many of the above mentioned works from other groups, the magnetic beads used in this study are much bigger commercially available magnetic beads. This can be favourable, as it has been shown in several studies [26,27,28] that with increasing size, the beads typically move faster when they are subjected to a magnetic gradient field. This behaviour enables prior sample enrichment by magnetic separation, leading to higher concentrations inside a smaller volume and therefore easier detection. The size distribution of magnetic beads is usually approximated by a lognormal function but due to agglomeration or mixture preparation, other distributions may occur that can be determined from measurements, for example, by means of a numerical inversion method [29].

## 2. Materials and Methods

### 2.1. Magnetic Beads

In this research, we used commercially available superparamagnetic beads from three different manufacturers. They had mean hydrodynamic diameters in the range between 70 nm to 1200 nm and were all functionalized with a streptavidin coating. This coating can be used, for example, to bind biotinylated antibodies to their surface to gain a target-specific labelling. As they are superparamagnetic, they exhibit a non-linear magnetization curve and no hysteresis [1]. This means that they have no remanent magnetization at zero applied field. 

We used the bead type MagSIGNAL-STA from MagnaMedics GmbH (Aachen, Germany) with a hydrodynamic diameter of 300 nm and an iron content of 10 mg/mL. In the following, they will be called 300nmMagSIGNAL. 

The 750 nm hydrodynamic diameter beads screenMAG/OP with an iron content of 10 mg/mL from chemicell GmbH (Berlin, Germany) are called 750nmChemicell in the following.

From micromod Partikeltechnologie GmbH (Rostock, Germany), we used their standard product nanomag-CLD with a hydrodynamic diameter of 500 nm and an iron content of 10 mg/mL (called here 500nmCLD). Additionally, beads with only 72 nm hydrodynamic diameter (iron content: 6 mg/mL, Lot: 104-19-701, called here 70nmSynomagD) and bigger ones with a hydrodynamic diameter of 865 nm (iron content: 6 mg/mL, Lot: 104-19-802, called here 800nmSynomagD) were used. Furthermore, the bead type (Article-No. 104-19-103 S04418, Lot: 044418104, iron content: 6 mg/mL) is used which is generated by an aggregation of the cores of their synomag beads. This type has a hydrodynamic diameter of 1188 nm (Z-Average, polydispersity index (PDI): 0.286) and is called 1µmSynomagD. The last bead type (Article-No. 05-19-502 S04718, Lot: 0471805, iron content: 6 mg/mL) is generated by a combination of their synomag and nanomag-CLD bead types during synthesis. These beads have a hydrodynamic diameter of 1010 nm (Z-Average, PDI: 0.227) and are called 1µmNanomag/SynomagCLD.

### 2.2. Principles of Magnetic Frequency Mixing Detection

**Figure 1:**
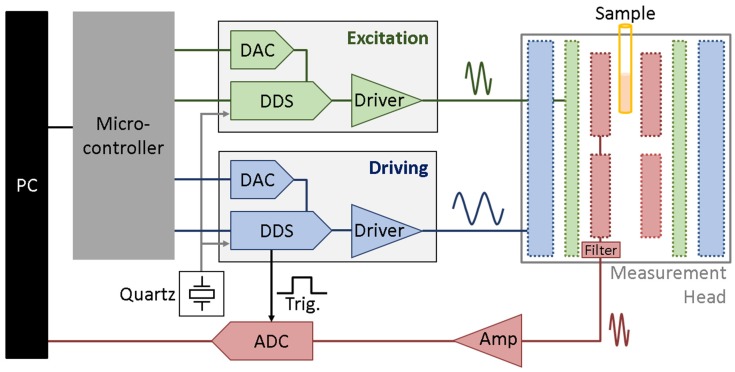
Schematic overview of the magnetic frequency mixing detection setup. A PC is controlling the magnetic reader which consists of a microcontroller, two direct digital synthesis (DDS) chips, digital to analogue converters (DAC), drivers, preamplifier and filters. A schematic cut of the measurement head is shown with the excitation (green) and driving (blue) field coil. Also, the sample (orange) inside one section of the differential detection coil (the measurement coil) is shown. The output of this detection coil is connected via filters and preamplifier (Amp) (inside the magnetic reader) to a measurement card. Additionally, there is a connection from the driving DDS of the magnetic reader to the analogue to digital converter (ADC) for a triggered recording.

Magnetic frequency mixing [12] is a technique for selectively detecting superparamagnetic beads in a sample. The sample containing MBs is placed inside a measurement head consisting of three coils, as shown in Figure 1. The two outer coils are for magnetic field generation, the innermost coil is the detection coil. The outermost coil is used for applying a magnetic driving field *f*_2_ with a frequency of a few tens of Hertz. The field’s amplitude is in the range of a few mT and therefore strong enough to drive the MBs in the nonlinear range of their magnetization curve at the extrema of the driving field. In this research, a frequency of 62.957 Hz was chosen. The middle coil is used to provide an excitation frequency magnetic field in order to probe the current magnetization state of the MBs. A magnetic field with a frequency *f*_1_ in the range between 11 and 82 kHz was used. If the beads are already magnetically saturated by the driving field *f*_2_, their response to the probing field is very weak. When the driving field is around zero, the probing field yields a strong response because it generates a strong alternating magnetization of the beads. Because of these two oscillating magnetic fields and the bead’s superparamagnetic behaviour, new frequencies are generated which follow the scheme: *f_new_* = *m*·*f*_1_ ± *n*·*f*_2_, where *m* and *n* are natural numbers. When there is no static magnetic offset field, the mixing components *f_new_* = *f*_1_ ± 2·*f*_2_ (*m* = 1, *n* = 2) have the highest signal amplitudes and are typically used for detection of the beads [12]. The innermost coil is used to detect these harmonics. The MBs, as well as the excitation fields, induce a signal in this coil. It is therefore built as a differential coil which means that it consists of two coils (called measurement and reference coil) which are connected in series. Both sections of this coil have identical parameters like diameter, length, number of turns but have a different winding direction. Due to this differential coil scheme, all magnetic fields that affect both coil sections the same way are cancelled out, like the very strong directly induced signals of the excitation field. As schematically shown in Figure 1, the magnetic beads are placed only in one part of the detection coil (called measurement coil), therefore their signal is picked up mainly just by this coil, so that it is not cancelled out.

### 2.3. Magnetic Frequency Mixing Detection Setup

Our setup consists of a so-called magnetic reader with measurement head connected with an analogue to digital converter measurement card from National Instruments (type NI USB-6251) and a PC. A simplified schematic representation of the setup is shown in Figure 1.

The PC runs custom software written in LabVIEW 2016. It controls the magnetic reader as well as the connected measurement card. 

The magnetic reader contains two synchronized direct digital synthesis (DDS) chips from Analog Devices (AD9834) connected to the same 50 MHz quartz for a stable phase relationship between them. Via serial peripheral interface (SPI), the frequencies and phases for driving and excitation field frequency DDS chips, as well as the amplitudes of these fields, can be set by individually connected digital to analogue converters (DAC). To ensure a fixed phase difference between both DDS chips, they are reset using the same pin of the microcontroller. During the experiments performed in this research, both DDS were operated with a phase difference set to 0°.

The inverted and non-inverted outputs of the DDS are used to generate an output, which’s offset value is not depending on the amplitude of the sine wave output. The signal is then changed to a bipolar output and current and voltage are amplified before they go to the respective coil.

The DDS chips have a 28-bit width frequency register. With the master frequency clock source of *f_MCLK_* = 50 MHz, the frequencies can be changed in steps of

(1) $$f_{Res}=\frac{f_{MCLK}}{2^{28}}=\frac{50\;\mathrm{MHz}}{2^{28}}=0.186\;\mathrm{Hz}.$$

The SignBit output of the driving frequency (*f*_2_) DDS, which changes its state when the most important bit of the internal DAC changes, is used for a triggered start of sample recording. In this research, we performed a direct digitization of the picked-up and preamplified signals, in contrast to prior work where a two-stage multiplication process was used [14,15,16,20]. This has the advantage that we can record many different harmonics at the same time and determine their phases without requiring a more complex hardware setup. When the measurement card is triggered, it records one million samples (1 MS) with a sampling frequency of 1 million samples per second (1 MSps). The measured values are transferred to the PC and a Fast Fourier Transformation (FFT) is performed which consequently has a frequency resolution of 1 Hz.

It can be shown (see Appendix A) that when the excitation frequency *f*_1_ is an integer multiple of the low driving frequency *f*_2_, also the mixing terms are integer multiples of the driving frequency. Because of this, a stable phase between *f*_1_ and *f*_2_ and using the same starting point in the trace of the *f*_2_’s wave leads to a stable phase information of the mixing frequencies.

### 2.4. Sample Preparation

In this study, we decided to use a simplified model system of a sandwich immunoassay. In such an assay, usually, the target is captured by an antibody which is fixed to a solid surface. On a different location of this target, a second capture molecule binds, for example, a secondary antibody. Then the label, in our case the MB, is attached to this compound. As the main purpose of our research is to study the distinction of different types of MB, we decided not to perform a complete sandwich immunoassay. Instead, the MBs were bound directly to a 3D immunofiltration column in order to suppress their Brownian movement. This is more like the situation in a sandwich immunoassay, as compared to a measurement in the liquid state. Additionally, this gives us the possibility of performing longer measurements without unfavourable side effects, for example, due to solvent evaporation or gravity-driven settling of the MBs. 

#### 2.4.1. Preparation of the Immunofiltration Columns

We used the same 3D immunofiltration columns (abicap HP columns, hydrophobic from Senova Gesellschaft für Biowissenschaften, Weimar, Germany), as have been used in different research [14,15,16], and fixed the MBs directly to the column’s surface.

Therefore, as a first step, the filters inside these columns need to be hydrophobically equilibrated. This was done by placing them in a beaker with ethanol in a desiccator and applying an underpressure to remove air from the pores of the filters. The columns were washed two times by applying 500 µL of distilled water on top of the filter and letting it flow through by gravity.

#### 2.4.2. Fixation of the Beads to the Surface of the Immunofiltration Columns

The MBs were immobilized on the surface by adding the MB solution to the 3D immunofiltration columns and letting it flow through by gravity flow. Afterwards, the columns were washed two times with 500 µL of distilled water. This is done to remove unbound MBs from the column. While in case of immunoassays, it is especially important to determine the concentration of a target without false positive readings, here it is most important not to have any changes of the MB’s positions in the filter during as well as between measurements. All given concentrations in this research are based on the concentration of the MBs inside their stock solution, as given by the manufacturer.

### 2.5. Measurement Procedure

#### 2.5.1. Performing the Measurements

The system together with the software for performing frequency scans was turned on and allowed to warm up for about half an hour until it has reached its equilibrium state. During this warm-up phase, the measurement process is already running and magnetic fields are generated by the coils inside the magnetic head. During this time, the system is heating up to the equilibrium temperature. After this warm-up phase, the sample is inserted and it is waited until the signals become stable and the system reaches an equilibrium state again. For example, due to the difference between room temperature and the temperature inside the measurement head, as well as possible hyperthermia effects, there are changes in the signal possible at the beginning. Afterwards, the frequency scan of the sample is started. During this scan, the excitation frequency *f*_1_ is changed from about 11.898 kHz to 81.781 kHz in steps of ten times the driving frequency *f*_2_, which is 62.957 Hz. Three measurements are performed at each value of the excitation frequency *f*_1_. There is a time delay of about two seconds between two consecutive measurements in order to verify that there is no change over time. This scanning was performed three times to make sure that the system was in equilibrium and that no changes occurred during the scan. During the frequency scans, the output amplitudes of the sinusoidal signals supplied to the excitation coils were not changed, therefore the resonance of the pickup circuit is clearly visible in the measured signals. To characterize the system, also scans without a sample in the measurement head, so-called background scans, were performed. In the amplitude region, these background scans were subtracted from the measurement signals acquired with sample in order to see more clearly the effect of the beads. An example is given in Supplementary Materials, Figure S1. After the scan, the system was put back to the state where no real scan is performed but the system was kept in the state as during a normal measurement, followed by equilibration and then scanning of the next sample.

#### 2.5.2. Data Processing and Handling

The measured data are saved as comma separated values in different files depending on the type of output, for example, amplitude and phase data. These data files were loaded into Origin 2015 (OriginLab Corporation, Northampton, MA, USA), plotted and further analysed. The amplitude and phase behaviour of the harmonic mixing component *f*_1_ + 2·*f*_2_ is given in this report.

## 3. Results and Discussion

### 3.1. Excitation Frequency Scan of Different MBs

**Figure 2:**
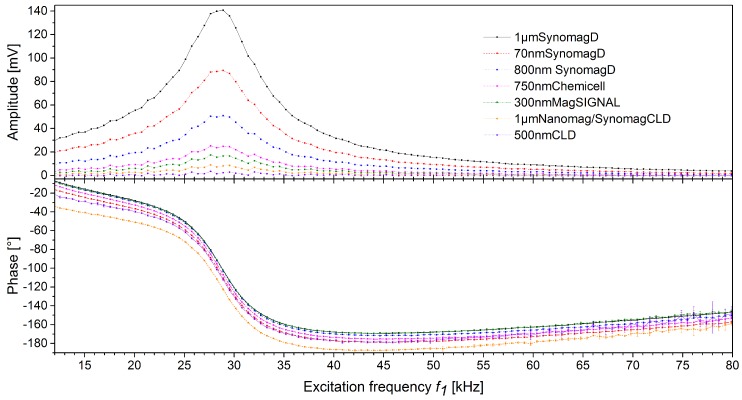
Amplitudes and phases of excitation frequency *f*_1_ scans (mean and standard deviation) with different magnetic bead (MB) types fixed in 3D immunofiltration columns. Numerical data to the plots are listed in Supplementary Materials, Table S1.

Different MBs were measured after fixation to a 3D immunofiltration column with various excitations frequencies *f*_1_ between 11.898 kHz to 81.781 kHz, as described in the “Materials and methods” section. It can be seen in Figure 2 that the amplitudes, as well as the phases, of all MBs are somewhat different but basically follow the same pattern. This pattern is dominated by the resonance of the measurement head and readout electronics. It can be seen that, for example, the phase of 1µmSynomagD (black) and 300nmMagSIGNAL (green) of different MBs are very similar while their amplitude is quite different.

### 3.2. Effect of Amount of MBs Fixed to the Filter

**Figure 3:**
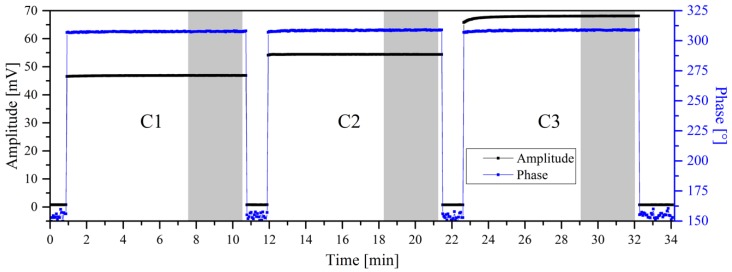
Signal trace while measuring three samples of 1µmSynomagD beads fixed to an immunofiltration matrix. The amount of bead solution used was 6, 8 and 10 µL (always diluted into 400 µL of distilled water), respectively and they were placed in the measurement head in that order. Shown is the signal trace of the amplitude and phase of the component *f*_1_ + 2·*f*_2_. The areas used for calculating the mean amplitude and phase of the samples (compare Table 1) are highlighted in grey. The excitation frequency (*f*_1_) was kept constant at 40.545 kHz during this experiment.

As already known, the amplitude signal is depending on the amount of MBs in the sample, which, for example, is used in References [14,15,16] to determine the target concentration in a sample. To verify this and to check if the phase of the frequency mixing signal depends on the concentration of the MBs immobilized on the filter, a fixed excitation frequency *f*_1_ of 40.545 kHz was used. This frequency was chosen because it is beyond the system’s resonance. The time trace of this experiment is shown in Figure 3. Here, three different amounts (6, 8, 10 µL bead solution +400 µL distilled water) of the same MB (1µmSynomagD from micromod) were fixed at the immunofiltration filter and measured one after another. A big difference between no sample and sample can be seen in the amplitude as well as in the phase traces. Among the different samples, a very strong response of the amplitude to the amount of beads used can be seen, whereas the phase remains almost unchanged. To quantify this effect, the mean values of the amplitude and the phase were calculated for each sample. The resultant values together with the mean and its standard deviation are listed in Table 1. The calculated values show that there is a strong response of the detected amplitude, which gets bigger when higher amounts of beads are used, whereas the phases are quite stable and no dependence on concentration can be observed.

### 3.3. Measurement of Samples Containing Two Different MBs

**Figure 4:**
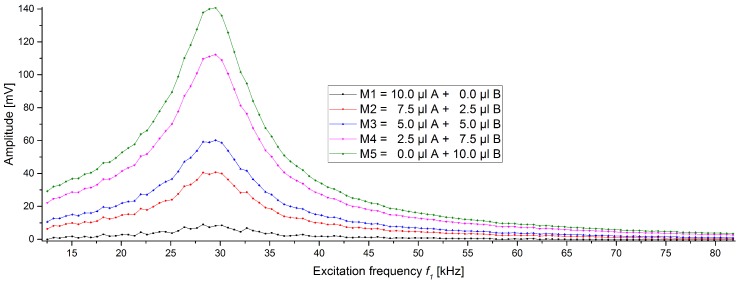
Amplitudes of frequency scans of 5 different samples containing different ratios of two beads (see Table 2). The total bead solution was always 10 µL in 400 µL of distilled water. Shown are the mean and standard deviations (within marker size).

**Figure 5:**
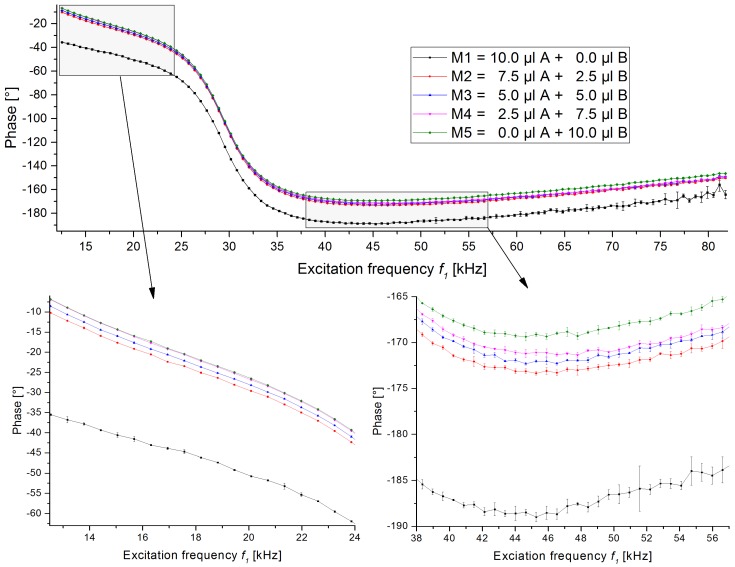
Phase traces of excitation frequency *f*_1_ scans of 5 different samples containing different ratios of two superparamagnetic beads (see Table 2). The total bead solution was always 10 µL in 400 µL of distilled water. The complete trace is shown in the top, while in the two bottom diagrams, the low frequency and the intermediate frequency areas are magnified. For each excitation frequency, the mean value and the standard deviation of the frequency mixing signal at *f*_1_ + 2·*f*_2_ are shown. In the bottom left graph, the traces of M4 and M5 overlap.

We prepared five mixtures with two different types of beads in which their proportion is varying between 0 and 10 µL. In this study, 1µmNanomag/SynomagCLD and 1µmSynomagD—both from micromod—were chosen as the two bead types. They were chosen because they are similar in size and therefore are expected to behave similarly in an immunoassay. They are named A and B, respectively. The mixtures are labelled as M1 to M5 and their contents are given in Table 2. The total volume of bead solution was always 10 µL and this volume was mixed into 400 µL of distilled water and flushed over the column. The amplitude and phase traces of these measurements can be seen in Figure 4 and Figure 5, respectively. In the case of the amplitudes, the background signal is subtracted from all scans.

The amplitudes (Figure 4) all follow the same basic pattern which also shows the resonance of our measurement system, as described before. It can be seen that 10 µL of 1µmNanomag/SynomagCLD (A, M1) shows the smallest signal response in these series, while the mixture with only 1µmSynomagD (B, M5) shows the highest signal for all frequencies used. The other mixtures lay between these two pure beads samples and their amplitudes follow the amount of 1µmSynomagD inside the sample (M1 → M5).

The phase traces of these scans are shown in Figure 5. As shown before in Figure 2, the phase traces of the pure beads (samples M1 and M5) can also be clearly distinguished. We assume that the main reason for the phase differences are different magnetic cores of both bead types. It has been shown for instance by Ludwig et al. [30] that the distribution of the core sizes influences the phase of the magnetic susceptibility. The phase traces of the mixtures are located between the traces of the pure beads and follow the trend of the bead’s proportion. The phases of the mixtures are closer to the phase of M5 than M1. For a better overview, zoomed sections are shown in the lower section of Figure 5. There we can see that in the region of low excitation frequencies *f*_1_ (Figure 5, bottom left), the phases of samples M4 and M5 show an overlap. In the intermediate excitation frequency region (Figure 5, bottom right), these samples are well separated but if the frequency is increased even further, the previously separated samples M3 and M4 are beginning to overlap.

### 3.4. Measurement of Mixtures with Amplitude Reduced MB Solution

It needs to be taken into account that in the amplitude behaviour, the bead’s basis solutions exhibit signals of different strength. Therefore, test measurements were performed where the bead solutions of 1µmSynomagD (B) which was generating higher signals was diluted to 7% of its original concentration ([B*] = 0.07·[B]), so that the amplitude difference among the various samples is not so strong anymore. The other bead type solution was kept the same (A* = A). All mixtures were prepared like in the previous test, as given in Table 2 but using this diluted stock solution instead of the manufacturer’s original one. All samples follow the same trend as before.

**Figure 6:**
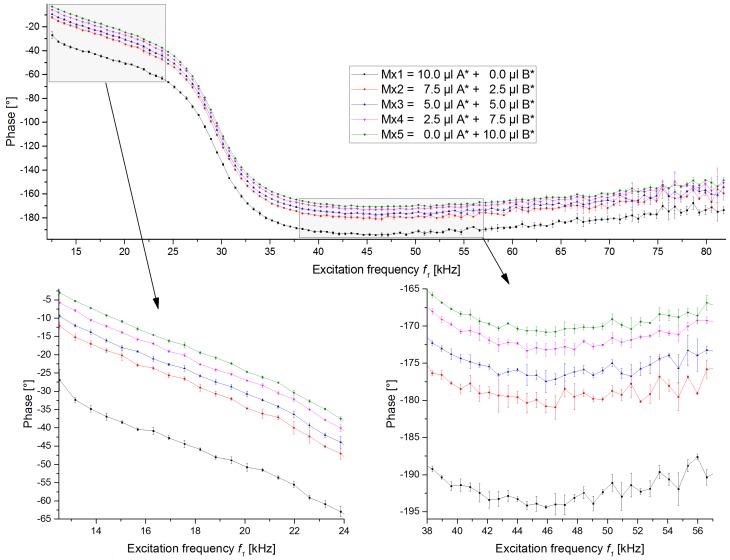
Phase traces of excitation frequency scans using the same beads and bead solution concentrations as in Figure 5/Table 2, except that the bead solution for SynomagD (bead B) is not the original manufacturer stock solution but an already diluted one, to reduce the dominant effect of this bead in the measurement. The mean and standard deviation at each excitation frequency are shown.

The recorded phases (Figure 6) for these mixtures follow the expected and previously described trend that the measured phase is following the proportion of the two bead types. The different samples show a difference of more than 20° in the low- and mid-frequency region for the pure bead samples, with the mixture samples in between, with differences of a few degrees. It can be seen very clearly that the different samples can be distinguished from each other based on the phase. In this scan, no overlaps of the samples are visible in the low- to mid-frequency regions. When the excitation frequency becomes larger, the signal starts to get less smooth and the signals start to overlap. This is in accordance to the non-diluted sample measurement.

## 4. Conclusions

As shown for samples containing different amounts of fixed MBs of the same kind, the amplitude changes with the amount. This effect is as expected, known from literature and is also widely used to determine the concentration of a target in an immunomagnetic sandwich assay using the magnetic frequency mixing technique. While the amplitudes change with the amount, the phases show no significant dependence on concentration. Therefore, it can be concluded that the amplitude is a good marker for the amount of beads inside a sample, whereas the phase is concentration-independent.

When comparing the phases of different MBs, it can be seen that the amplitudes as well as the phases deviate from each other. By generating different mixtures with varying proportions of two different types of MBs, it can be seen that not only the amplitudes change but also the phases. As it has been shown that the phase does not depend on the amount of beads, it can be concluded that the measured phase is affected by both bead types and therefore cannot only be used to determine the type of MB but also, in combination with the amplitude, to determine the amounts of the different types of MBs in one sample. 

Different amplitudes and phases were observed by changing the excitation frequency. By observing the phase behaviour of the mixtures, frequency regions were identified where the samples are well separable, while their signals overlap at other frequency regions. To be able to distinguish between different MBs and to get a good resolution, it might be preferable not only to use one fixed excitation frequency but to also vary it. Depending on different parameters, like the method used for the detection of the amount of MBs, the needed resolution and usable timeframe, the frequency steps in which the high excitation frequency is varied, and its lower and upper limit can be adapted.

## Figures and Tables

**Table 1:** Overview of the measured amplitudes and phases for three samples with different concentrations of 1µmSynomagD beads fixed to an immunofiltration matrix. Shown are the results for the component *f*_1_ + 2·*f*_2_. The excitation frequency (*f*_1_) was kept constant at 40.545 kHz during this experiment.

Bead Solution		Amplitude		Phase	
Name	Volume [µL]	Mean [mV]	Standard Deviation [mV]	Mean [°]	Standard Deviation [°]
C1	6	46.88	0.008	307.7	0.231
C2	8	54.39	0.009	308.8	0.245
C3	10	68.07	0.014	308.8	0.211

**Table 2:** Amount of the two bead types in samples M1 to M5. The total volume of the bead solution was always 10 µL pipetted into 400 µL of distilled water.

			Mixture				
			M1	M2	M3	M4	M5
**Bead Type**	A	1µmNanomag/Synomag CLD	10 µL	7.5 µL	5 µL	2.5 µL	0 µL
	B	1µmSynomagD	0 µL	2.5 µL	5 µL	7.5 µL	10 µL

## Supplementary Materials

The following are available online at https://www.mdpi.com/1424-8220/19/11/2599/s1, Figure S1: Measured amplitude as a function of excitation frequency *f*_1_ of 1µmSynomagD fixed in 3D immunofiltration columns, background scan and difference, Table S1: Data of the figures.

## Appendix A

In this section, we mathematically prove that the mixing frequencies’ (MFs) period fits as a positive integer multiple in the low frequency (*LF*, *f*_2_) period if the high frequency (*HF*, *f*_1_) period fits as a positive integer multiple in the low frequency (*LF*, *f*_2_) period.

The mixing frequencies (MFs) are defined as

(A1) $$f_{MF}=f_{HF}\pm a\cdot f_{LF},\;\mathrm{with}\;a\in N$$

and

(A2) $$f=\frac{1}{T},\;\mathrm{with}\;f=\mathrm{Frequency}\;[\mathrm{Hz}]\;\mathrm{and}\;T=\mathrm{Period}\;[1/s].$$

Using Equation (A2) in (A1) leads to

(A3) $$\frac{1}{T_{MF}}=\frac{1}{T_{HF}}\pm a\cdot \frac{1}{T_{LF}}$$

This can be solved for *T_MF_* as the following:

(A4) $$T_{MF}=\pm \frac{T_{HF}\cdot T_{LF}}{a\cdot T_{HF}\pm T_{LF}}$$

As it was stated in used frequencies of our application: The *HF* is chosen as an integer multiple of the *LF*

(A5) $$f_{HF}\;\mathrm{mod}\;f_{LF}=0,$$

which can also be written as

(A6) $$T_{HF}\cdot b=T_{LF},\;\mathrm{with}\;b\in N$$

or

(A7) $$T_{HF}=\frac{T_{LF}}{b}.$$

Applying Equation (A7) in (A4) leads to

(A8) $$T_{MF}=\frac{\frac{T_{LF}}{b}\cdot T_{LF}}{a\cdot \frac{T_{LF}}{b}\pm T_{LF}}.$$

This can be written in short as

(A9) $$T_{MF}=\frac{T_{LF}}{b\pm a}.$$

Solving (A9) and (A6) for *T_LF_* leads to

(A10) $$T_{LF}=T_{MF}\cdot \left(b\pm a\right)$$

and

(A11) $$T_{LF}=T_{HF}\cdot b.$$

Analysing the (b±a) term in A11 depending on the sign:

- b+a: as *a*, *b* are element of ℕ also there sum is an element of ℕ: (*b* + *a*) ∈ ℕ.
- b−a: as *a*, *b* are element of ℕ also there sum is an element of ℕ if *b* > *a*: (*b* − *a*) ∈ ℕ, if *b* > *a*.

As in our application, the *HF* is always bigger than the *LF* (*b* > *a*) and therefore the term in the bracket is always positive, it can be concluded that the periods of all mixing frequencies’ periods fit as integer multiples inside the low frequency’s period.

This results in a stable phase difference between the mixing terms status with respect to the low frequencies status.
