# Supplementary material for: Multiplex Detection of Different Magnetic Beads Using Frequency Scanning in Magnetic Frequency Mixing Technique
Source: Sensors (Basel). 2019 Jun 7;19(11):2599. doi: 10.3390/s19112599 (PMC6603599; doi:10.3390/s19112599)
Supplement: Supplementary file 1 [file sensors-19-02599-s001.zip › sensors-516331-supp/sensors-516331-suppl-figure.pdf]

Supplementary Materials:

Article

Multiplex Detection of Different Magnetic Beads Using Frequency Scanning in Magnetic Frequency Mixing Technique

Stefan Achtsnicht [1, 2], Ali Mohammad Pourshahidi [1,2], Andreas Offenhäusser [1,2] and Hans-Joachim Krause [1,*]

[1] Institute of Complex Systems Bioelectronics (ICS-8), Forschungszentrum Jülich, 52425 Jülich, Germany; s.achtsnicht@fz-juelich.de (S.A.); a.pourshahidi@fz-juelich.de (A.M.P.); a.offenhaeusser@fz-juelich.de (A.O.)

[2] RWTH Aachen University, 52062 Aachen, Germany;

* Correspondence: h.-j.krause@fz-juelich.de; Tel.: +49-2461-61-2955

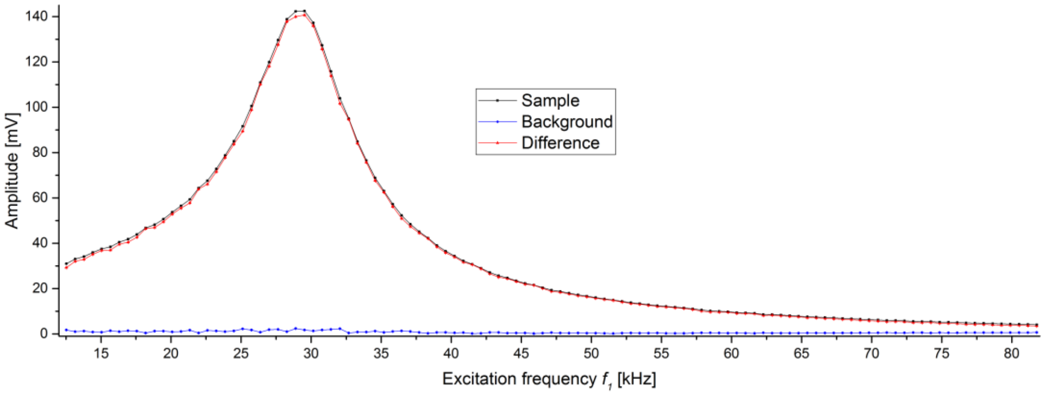

Figure 1. Measured amplitude as a function of excitation frequency f_1 of 1µmSynomagD fixed in 3D immunofiltration columns, background scan and difference.
